# Impact of Coffee, Wine, and Chocolate Consumption on Cognitive Outcome and MRI Parameters in Old Age

**DOI:** 10.3390/nu10101391

**Published:** 2018-10-01

**Authors:** Sven Haller, Marie-Louise Montandon, Cristelle Rodriguez, François R. Herrmann, Panteleimon Giannakopoulos

**Affiliations:** 1 CIRD—Centre d’Imagerie Rive Droite, Rue Chantepoulet 21, 1201 Genève, Switzerland; 2 Department of Surgical Sciences, Radiology, Uppsala University, 751 85 Uppsala, Sweden; 3 Faculty of Medicine, University of Geneva, 1205 Genève, Switzerland; 4 Department of Psychiatry, Faculty of Medicine, University of Geneva, 1205 Genève, Switzerland; mlmontandon@hotmail.com (M.-L.M.); Panteleimon.Giannakopoulos@hcuge.ch (P.G.); 5 Division of Institutional Measures, Medical Direction, University Hospitals of Geneva, Rue Gabrielle-Perret-Gentil 4, 1205 Genève, Switzerland; Cristelle.Rodriguez@hcuge.ch; 6 Division of Geriatrics, Department of Internal Medicine, Rehabilitation and Geriatrics, University of Geneva, 1205 Genève, Switzerland; Francois.Herrmann@hcuge.ch

**Keywords:** caffeine, wine, chocolate, aging, cognition

## Abstract

Coffee, wine and chocolate are three frequently consumed substances with a significant impact on cognition. In order to define the structural and cerebral blood flow correlates of self-reported consumption of coffee, wine and chocolate in old age, we assessed cognition and brain MRI measures in 145 community-based elderly individuals with preserved cognition (69 to 86 years). Based on two neuropsychological assessments during a 3-year follow-up, individuals were classified into stable-stable (52 sCON), intermediate (61 iCON) and deteriorating-deteriorating (32 dCON). MR imaging included voxel-based morphometry (VBM), tract-based spatial statistics (TBSS) and arterial spin labelling (ASL). Concerning behavior, moderate consumption of caffeine was related to better cognitive outcome. In contrast, increased consumption of wine was related to an unfavorable cognitive evolution. Concerning MRI, we observed a negative correlation of wine and VBM in bilateral deep white matter (WM) regions across all individuals, indicating less WM lesions. Only in sCON individuals, we observed a similar yet weaker association with caffeine. Moreover, again only in sCON individuals, we observed a significant positive correlation between ASL and wine in overlapping left parietal WM indicating better baseline brain perfusion. In conclusion, the present observations demonstrate an inverse association of wine and coffee consumption with cognitive performances. Moreover, low consumption of wine but also moderate to heavy coffee drinking was associated with better WM preservation and cerebral blood-flow notably in cognitively stable elders.

## 1. Introduction

Coffee, wine and chocolate are three frequently consumed substances with a significant impact on cognitive performances.

Early studies in community-based samples suggested that moderate caffeine consumption is associated with decreased incidence of both mild cognitive impairment (MCI) and clinically overt AD [1,2,3,4]. More recently, a case-control study revealed that plasma caffeine levels greater than 1200 ng/mL in MCI subjects were associated with no conversion to dementia during a 2–4-year follow-up [5]. Importantly, in the Italian Longitudinal study of aging, moderate caffeine consumption over time (from 1 to 2 cups of coffee/day) was associated with lower incidence rate of MCI in cognitively intact older individuals. However, an inverse association was found for those who increased their daily caffeine consumption [6,7].

A U-shape relationship between cognitive performance and wine consumption has been postulated with a marked detrimental effect of heavy drinking but a decrease of Alzheimer disease (AD) and dementia risk among light to moderate drinkers. However, this latter association has been challenged due to confounding by socioeconomic class and intelligence (for review see References [8,9,10]).

Recent lines of evidence suggest that regular consumption of cocoa is associated with dose-dependent improvements in general cognition, attention, processing speed, and working memory that have been documented in animal models of normal aging but also in a limited series of healthy elders (for review see [11,12]).

The impact of these substances on resting state brain function and AD pathology has been intensively explored. A limited number of randomized controlled trials explored the acute effects of caffeine, cocoa flavonoids and alcohol in brain function and perfusion [13,14,15]. Overall, caffeine intake was associated with a significant reduction of ASL-measured gray matter cerebral blood flow, increased load-related activation compared to placebo in the left and right dorsolateral prefrontal cortex during working memory encoding, but decreased load-related activation in the left thalamus during working memory maintenance. Alcohol intake led to increased cerebral blood flow in a dose-dependent manner (for review see Joris et al. [16]). Chronic caffeine intake has been shown to reduce Aβ-induced cell death in vitro, decrease brain amyloid levels [6,17,18,19,20,21], reduce hippocampal tau phosphorylation and proteolytic fragments but also mitigate several proinflammatory and oxidative stress markers in AD transgenic models [22]. Several studies pointed to a caffeine-mediated decrease of resting-state connectivity across the brain in healthy controls. More recently, it was shown that although this is true in respect to visual and motor areas, the blood oxygenation level dependent (BOLD) functional connectivity of the default mode network (DMN) might increase via the recruitment of attentional networks partly explaining the caffeine-mediated elevated alertness [23,24,25,26]. Low concentrations of ethanol have been shown to protect against toxicity induced by Aβ oligomers [27]. In alcohol drinkers (without misuse or dependence), resting state functional connectivity is reduced in posterior cortical areas as precuneus, postcentral gyrus, insula, right fusiform and lingual gyri and visual cortex [28] but also in the sub-callosal cortex, in left temporal fusiform cortex and left inferior temporal gyrus [29]. In the same line, cocoa extracts reduce oligomerization of beta amyloid and modulates the brain neurotrophic-derived factor signalling pathway in AD animal models [30,31]. At the cellular level, chocolate and other flavonoids interact with signalization cascades involving protein and lipid kinases that lead to the inhibition of neuronal death by apoptosis induced by neurotoxicants such as oxygen radicals and promote neuronal survival and synaptic plasticity (for review see [32]).

Contrasting with the substantial amount of data on resting state fMRI effects of wine, coffee and chocolate intake, a surprisingly low number of studies addressed the consequences of their chronic consumption on structural MRI parameters in healthy controls (without any misuse or addiction-related behaviors). Most of them concerned alcohol beverages and remain highly controversial. Linear decrease of grey matter (GM) volumes were reported with weekly alcohol consumption mainly in men whereas white matter (WM) volume analysis led to conflicting data [33,34,35,36]. Regular caffeine use is known to reduce arterial spin labelling (ASL)-assessed cerebral blood flow (CBF) [37,38] in healthy controls. To our knowledge, there were no studies investigating the relationship between chocolate consumption and structural MRI parameters as well as ASL-assessed CBF.

In order to define the structural and cerebral blood flow correlates of regular consumption of coffee, wine and chocolate in old age, we performed voxel-based morphometry (VBM), tract-based spatial statistics (TBSS) that detect changes in grey and white matter microstructure and arterial spin labelling (ASL) perfusion imaging in a community-based series of 145 elderly individuals aged from 69.3 to 85.8 who were cognitively preserved at inclusion and underwent two neuropsychological assessments during a subsequent 3-year period.

## 2. Materials and Methods

### 2.1. Participants

The data engaged in this article was retrieved from an ongoing large population-based longitudinal study on healthy aging that is still ongoing in the Geneva and Lausanne counties. The cohort included 526 elderly Caucasian white individuals living in Geneva and Lausanne catchment area. Due to the need for excellent French knowledge (in order to participate in detailed neuropsychological testing) the vast majority of the participants were Swiss (or born in French-speaking European countries, 92%). At baseline, all individuals were evaluated with an extensive neuropsychological battery, including the Mini-Mental State Examination (MMSE) [39], the Hospital Anxiety and Depression Scale (HAD [40]), and the Lawton Instrumental Activities of Daily Living (IADL, [41]). Cognitive assessment included (a) attention (Digit-Symbol-Coding [42], Trail Making Test A [43]), (b) working memory (verbal: Digit Span Forward [44]), visuo-spatial: Visual Memory Span (Corsi) [45], (c) episodic memory (verbal: RI-48 Cued Recall Test [46]), visual: Shapes Test [47], (d) executive functions (Trail Making Test B [43], Wisconsin Card Sorting Test and Phonemic Verbal Fluency Test), (e) language (Boston Naming [48]), (f) visual gnosis (Ghent Overlapping Figures), (g) praxis: ideomotor [49], reflexive [50], and constructional (Consortium to Establish a Registry for Alzheimer’s Disease (CERAD), Figures copy [51]). All individuals were also evaluated with the Clinical Dementia Rating scale (CDR) [52]. In agreement with the criteria of Petersen et al. [53], participants with a CDR of 0.5 but no dementia and a score exceeding 1.5 standard deviations below the age-appropriate mean in any of the cognitive tests were classified as MCI and were excluded. Participants with neither dementia nor MCI were classified as cognitively healthy controls and underwent full neuropsychological assessment at follow-ups, on average 18 and 36 months later. Exclusion criteria included psychiatric or neurologic disorders, sustained head injury, history of major medical disorders (neoplasm or cardiac illness), alcohol or drug abuse, regular use of neuroleptics, antidepressants or psychostimulants and contraindications to MR imaging. To control for the confounding effect of cardiovascular diseases, individuals with subtle cardiovascular symptoms and a history of stroke, severe hypertension and transient ischemic episodes were also excluded from the present study.

At follow-up, which took place 18 months after inclusion, the cognitively healthy individuals underwent full neuropsychological assessment. Individuals who obtained stable cognitive scores over the baseline and follow-up evaluation were classified as stable controls. The progressive control group obtained a follow-up evaluation of at least 0.5 standard deviations (SD) lower than measured at baseline, on a minimum of two cognitive tests. Two neuropsychologists clinically assessed all individuals independently. The final classification was determined by a trained neuropsychologist considering both the results of the neuropsychological tests and overall clinical assessment [54]. All of the case’s individuals were assessed once again 18 months later with the same neuropsychological battery. The participants were subsequently grouped as described above (−0.5 SD in at least two cognitive tests), with comparison of the scores of the latest assessment. Stable individuals showing no changes in the second assessment were classified in the stable-stable (sCON) group and progressive individuals demonstrating a further decline as deteriorating-deteriorating (dCON). The intermediate group (iCON) refers to participants demonstrating a fluctuating scoring pattern, incorporating stable-progressive, progressive-stable or progressive-improved individuals.

The final sample consisted of 52 sCON (mean age 73 ± 3 years; 32 women), 61 iCON (mean age 73 ± 3 years; 30 women) and 32 dCON (mean age 74 ± 4.0 years; 18 women). All participants gave informed written consent after formal approval by the local Ethics Committee.

The timeline of neuropsychological assessment, MR imaging and questionnaire is illustrated online in Figure S1.

### 2.2. Substance Questionnaire

Usual caffeinated foods and beverages (coffee, chocolate) consumption as well as wine intake were assessed by a self-administered questionnaire. Participants were asked to complete the questionnaire entering the amount consumed by day, month and year (see online Supplementary Material). After reception of the questionnaire and in case of doubt, additional information was obtained by phone calls in order to obtain a global estimation of the consumption. In contrast, the type of coffee preparation or wine was not explored further since no lines of evidence indicate a differential impact of these preparations (or type of wine) in the human brain. The caffeine questionnaire was derived from Reference [55] and related caffeine content can be found in References [56,57].

### 2.3. MRI Data Acquisition

Imaging data were acquired on a 3T MRI scanner (TRIO SIEMENS Medical Systems, Erlangen, Germany) Essential data include: a high-resolution T1-weighted anatomical scan (magnetization prepared rapid gradient echo (MPRAGE), 256 × 256 matrix, 176 slices 1 mm isotropic, TR = 2.27 ms), a pulsed ASL sequence (64 × 64 matrix, 24 slices, voxel size 3.44 × 3.44 × 5 mm^3^, TE = 12 ms, TR = 4000 ms, inversion time (TI) 1600 ms) and a diffusion tensor imaging DTI sequence (b = 0 and 30 diffusion directions with *b* = 1000 s/mm^2^, 128 × 128 matrix, voxel size 2.0 × 2.0 × 2.0 mm^3^, TE = 82.4 ms, TR = 7900 ms and 1 average).

Additional sequences included axial fast spin-echo T2w imaging (4000/105, 30 sections, 4-mm section thickness), susceptibility weighted imaging (28/20, 208 × 256 × 128 matrix, 1 mm × 1mm × 1 mm voxel size) were performed to exclude brain disease, such as ischemic stroke, subdural hematomas, or space-occupying lesions.

### 2.4. Statistical Analysis of Demographic and Substance Data

Comparison among the three groups were performed with Fisher exact test, Kruskal-Wallis test or one way ANOVA according to the distribution of the variables. Caffeinated foods and beverages were considered as continuous variables, *z*-scores and also as tertile (light, moderate, heavy consumers). Consumption of coffee was divided in tertile as follows: light (0–28 cups/month), moderate (29–60 cups/month), heavy (61–168 cups/month). Light drinkers for wine corresponded to a consumption of 0–8 units /month, moderate to a consumption of 9–28 units /month, and heavy to a consumption of 29–200 units/month. Consumption of chocolate was divided in tertile as follows: light (0–20 serving/month), moderate: 20–80 serving/month, heavy: 81–226 serving/month). Unadjusted, adjusted and multiple ordered logistic regression models were used to predict group membership (see results section for details) from the different type of consumptions (chocolate, coffee and wine).

### 2.5. MR Data Analysis

#### 2.5.1. Whole-Brain Voxel-Based Morphometry (VBM)

The voxel-based morphometry analysis was carried out using the FSL software package [58], according to the standard procedure. The essential processing steps included brain extraction using Brain Extraction Tool [59], tissue-type segmentation using FMRIB’s Automated Segmentation Tool [60], nonlinear transformation into Montreal Neurological Institute (MNI) reference space, and creation of a study-specific GM template to which the native GM images were then nonlinearly re-registered. The modulated segmented images were then smoothed with an isotropic Gaussian kernel with a sigma of 2 mm. Finally, the voxel-wise FSL General Linear Model was applied by using permutation-based non-parametric testing with the FSL Randomize Tool with the threshold-free cluster enhancement (TFCE) correction for multiple comparisons [61], considering fully corrected *p* values < 0.05 as significant. The analysis was performed twice. First, the analysis was performed across all participants across the entire brain using coffee, wine or chocolate as dependent variables- and age, gender, education and MMSE score as potential confounders. Second, the analysis was performed as separate models for the groups sCON, iCON and dCON using only one explanatory variable (coffee, wine or chocolate) and again age, gender, education and MMSE score as non-explanatory variables.

#### 2.5.2. Arterial Spin Labelling (ASL)

The reconstructed relCBF (relative cerebral blood flow) ASL perfusion images were spatially normalized using a linear spatial alignment from ASL raw data to the individual high-resolution 3DT1 image, followed by the application of the non-linear spatial registration determined in the pre-processing of the 3DT1 data. The spatial transformations were then applied to the relCBF maps calculated directly on the MRI scanner, this two-steps approach results in a non-linear spatial registration of the ASL relCBF map into the MNI space. We then calculated the whole brain average relCBF, which was compared between groups with caffeine, wine and chocolate as dependent variables with age, gender, education and MMSE score as potential confounders. Moreover, we applied a voxel-wise local permutation-based, with threshold-free cluster enhancement (TFCE) correction for multiple comparisons, considering fully corrected *p* values < 0.05 as significant. The statistical models were performed similar to VBM described above.

#### 2.5.3. Diffusion Tensor Imaging (DTI) Tract Based Spatial Statistics (TBSS)

The TBSS analysis of the DTI data was done implementing the FSL software package [58], according to the standard procedure described in detail [62]. All subjects’ FA data were projected onto a mean FA skeleton using a non-linear spatial registration. The tract skeleton is the basis for voxel-wise cross-subject statistics and reduces potential misregistrations as the source for false-positive or false-negative analysis results. The other DTI-derived parameters—longitudinal, radial, and mean diffusivity were analyzed in the same way using spatial transformation parameters that were estimated in the initial FA analysis. Similar to the VBM analysis above, the TBSS was analyzed using voxel-wise statistical analysis was performed TFCE correction for multiple comparisons, considering fully corrected *p* values < 0.05 as significant. We used the John Hopkins University DTI-based white matter tractography atlas, which is distributed in the FSL package, for anatomic labeling of the supra-threshold voxels. The statistical models were performed similar to VBM described above.

#### 2.5.4. GM Region of Interest (ROI) Analysis

In addition to the voxel-wise whole-brain analysis described above, we additionally performed a region of interest (ROI) analysis. The whole was parcellated into 133 regions using the Combinostics cMRI software package [63]. We performed bivariate linear regression models to predict each MRI regional parameters from group and each substance entered either as *z*-score or as an ordinal variable (tertile).

## 3. Results

### 3.1. Clinical, Demographic and Substance Data

The clinical and demographic data are summarized in Table 1. There were no statistically significant differences in age, gender and education among the groups sCON, iCON and dCON.

When including one type of consumption as z-score in ordered logistic regression model to predict group membership without and while adjusting for age, sex, education level and MMS, only wine was associated with an increased risk of adverse evolution (OR_unadjusted_ 1.012, 95% CI 1.002–1.023; *p* = 0.017 unadjusted), (OR_adjusted_ 1.012, 95% CI 1.001–1.022; *p* = 0.028 adjusted). In a multiple ordered logistic regression model adjusted for the same confounders as above and all type of consumptions, wine consumption remained significantly associated with the dCON status (OR_adjusted_ 1.401, 95% CI 1.003–1.955; *p* = 0.048).

When analyzing the consumption data as tertile, moderate coffee drinkers are less likely to be classified as dCON (OR_unadjusted_ 0.451, 95% CI 0.214–0.950; *p* = 0.036) (OR_adjusted_ 0.447, 95% CI 0.210–0.952; *p* = 0.037). This observation persists after adjusting for wine and chocolate consumption OR_adjusted_ = 0.455; 95% CI 0.208–0.995; *p* = 0.048.

### 3.2. MRI Analysis across the Entire Group

**Figure 1:**
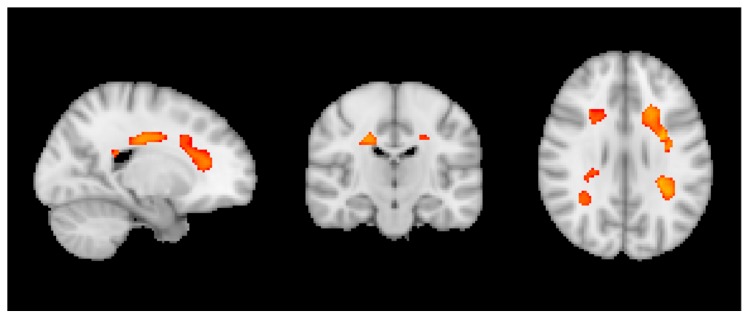
Negative correlation between wine and VBM across all individuals. *p* < 0.05 TFCE corrected.

Across all participants, we observed a negative correlation in VBM with wine notably in bilateral deep white matter regions (Figure 1).

In contrast, no significant differences were observed for ASL or TBSS measures as a function of the substances studied.

### 3.3. Group MRI Analysis 

**Figure 2:**
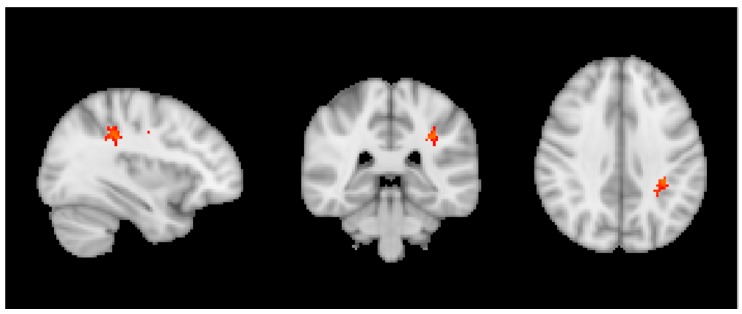
Positive correlation between wine and ASL for only sCON individuals. *p* < 0.05 TFCE corrected.

In sCON cases, we observed a significant positive correlation between ASL measures and wine in left parietal white matter (Figure 2), overlapping with the results of the VBM correlation of all individuals reported above.

**Figure 3:**
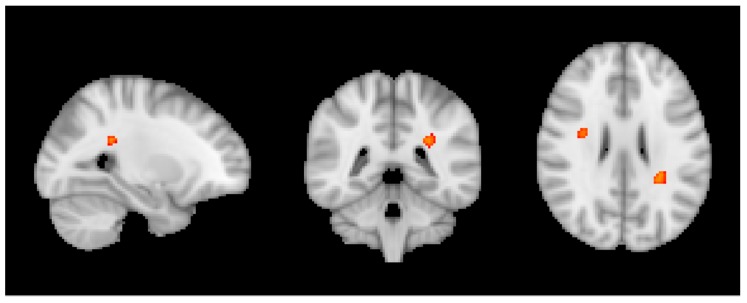
Negative correlation between caffeine and VBM for only sCON individuals. *p* < 0.05 TFCE corrected.

Moreover, we observed a negative correlation between VBM and caffeine only in sCON individuals notably in the white matter that was more pronounced in left parietal and right frontal regions (Figure 3).

Importantly, there were no significant associations between these substances and MRI findings in both iCON and dCON groups.

## 4. Discussion

We demonstrate an inverse association of wine and coffee consumption with cognitive performances. In addition, low consumption of wine but also moderate to heavy coffee drinking was associated with better WM preservation and cerebral blood-flow notably in cognitively stable elders.

At the behavioral level, the present study reveals that moderate consumption of caffeine is related to better cognitive outcome in a community-based sample of 145 elderly controls that undergo two detailed neuropsychological follow-ups in a 3-year period. Importantly, this association is limited to low quantities and did not persist in cases with very subtle signs of cognitive instability (iCON) or early phases of cognitive decline (dCON).

In contrast, increased consumption of wine is related to unfavorable cognitive evolution. The relationship between drinking and cognitive performances in old age remains a highly controversial issue. The deleterious effect of heavy wine consumption on cognitive evolution over time in elderly controls has been already documented [8,9,10,64,65]. Several lines of evidence have suggested that moderate drinking could have a slight positive impact on memory and verbal abilities [66,67] but negative data have been also reported [64,68]. In our highly selected cases that mostly consumed very low levels of alcohol (more than 75% among them consumed less than one unit per day and almost one third less than eight units per month), we failed to document a positive association between moderate wine drinking and cognition. In contrast, we found a negative relationship between increased wine consumption and neuropsychological performances as already suggested previously (for review see Reference [64]). It should, however, be noted that this finding was obtained when using z-scores but not tertiles indicating that the heavy consumption of a limited number of elderly controls led to this result. In contrast to wine, moderate caffeine consumption (up to two cups of coffee/day) was associated with better cognitive outcome in our 3-year follow-up. This observation parallels several previous reports on the protection conferred by moderate caffeine consumption in cognitive aging [1,2,3,4,5]. Not surprisingly, chronic chocolate consumption was not associated with cognition in our elderly controls. A positive effect of cocoa products seems to be confined to acute consumption as previously reported [11,12].

Concerning brain MRI, we first assessed the entire dataset of healthy elderly controls and observed a negative correlation between wine consumption and VBM in bilateral fronto-parietal white matter (WM). This result may appear contra-intuitive at first glance, as VBM is usually used to assess modifications in grey matter (GM) concentration. However, it should be noted that microvascular WM lesion are very frequent in the elderly population. They appear as hypersignal on T2w/FLAIR (fluid attenuated inversion recovery) sequences, and are usually reported on those sequences, e.g., using the Fazekas score. Although less evident and consequently usually less frequently assessed, those microvascular WM lesions also appear as a hypointense signal on T1w images, which is the basis of the VBM analysis. The negative correlation between wine and VBM in WM indicates less hypointense signal on T1 and consequently a reduced severity of WM lesions with increasing wine intake. Interestingly, the additionally performed TBSS analysis of the WM skeleton did not reveal significant differences in FA (fractional anisotropy), which is considered as a microstructural marker of axonal integrity. Taking together the results of VBM and TBSS, this indicates that increased wine intake may reduce microvascular lesions of the fronto-parietal WM, while association between this consumption and microstructural integrity of the WM seems more difficult to establish. Interestingly, an increasing number of studies point to the positive association between low to moderate wine consumption and WM integrity. In particular, Verbaten reported less white matter damage in elderly light and moderate drinkers [33]. Similar results were reported by Mukamal for elders consuming less than six units per week [69] for the vast majority of the present cases. Interestingly and unlike cognitive performances, we did not detect a negative association between heavy drinking and WM integrity. The absence of a U-shape association here may be related to the limited number of heavy drinkers in this sample and low exposure to cardiovascular risk factors due to the exclusion criteria.

A separate set of findings concerned with the association between consumption and brain structure as a function of the cognitive fate in this longitudinal series. We built regression models for each subgroup. Based on repeated neurocognitive testing, the healthy controls were sub-classified into sCON, iCON and dCON. It is important to emphasize that even for the dCON participants, the cognitive profile remains within the normal limits at follow-up, however, the individual cognitive profile slightly decreased two times at 18 and 36 months follow-up. In contrast, the cognitive profile remains constant twice for the sCON participants, and is intermediate for the iCON participants. Only in the sCON individuals, we observed a positive correlation between wine and ASL in the WM, overlapping with the regions of the VBM results across all participants reported above. This indicates that wine does not only reduce the WM lesion load, but also improves brain perfusion at baseline; however, this effect is limited in cases who remained cognitively stable over time. It is noteworthy that among sCON cases, only six cases corresponded to the classical definition of heavy drinking (≥8 units for women and 15 for men), the mean consumption being less than one unit/day. In the same line, we found a negative association between caffeine consumption and VBM only for sCON participants in the right frontal and left parietal WM regions, without a significant association with TBSS parameters. Similar to the argumentat above, this might indicate that caffeine reduces WM lesion load only in sCON participants, without having a significant effect on WM microstructural integrity. Interestingly, and in contrast to wine, most of the sCON cases were of moderate or heavy consumption of caffeine, not supporting the idea of a U-shaped association between caffeine consumption and WM lesions. Moreover, the positive association between caffeine consumption and cognition was present only in sCON participants consistent with the view that caffeine is a cognitive normalizer rather than a cognitive enhancer [70,71]. As for cognitive outcome, chocolate consumption was not associated with the MRI parameters studied in the present series suggesting that the chronic consumption of chocolate is not beneficial nor deleterious for brain integrity or cognitive performances in old age.

## 5. Conclusions

In conclusion, the present observations confirm the opposite associations between wine and coffee consumption on cognitive performances, suggesting a detrimental effect of heavy drinking and benefits of chronic consumption of moderate quantities of coffee. The low consumption of wine but also moderate to heavy coffee drinking is associated with better WM preservation and cerebral blood-flow in cognitively stable elders without significant cerebrovascular pathologies. Strengths of the present study include the longitudinal follow-up with detained neuropsychological battery in all of our community-dwelling cases and absence of health-related confounders such as neurological, psychiatric and cerebrovascular pathologies. Several limitations should however be considered when interpreting these data. First, our cohort of healthy controls was without significant vascular pathology and a high level of daily functioning without any symptom of substance abuse is not representative of the entire spectrum of old age. Second, current consumption was assessed with a food questionnaire based on self-reporting, leading to possible underestimation of wine consumption. Third, no data on lifetime consumption were obtained, so the possible deleterious or beneficial effect of wine and coffee use at midlife cannot be assessed. Finally, MRI assessment was performed at baseline and thus we cannot comment on the association between MRI structural parameter changes and wine and coffee consumption over time. Future studies in large community-based samples combining self and proxy-reports, lifetime assessment of wine and coffee consumption and repeated MRI scans are needed to shed additional light into the complex relationships between these substances and structural MRI parameters in old age.

## Figures and Tables

**Table 1:** Clinical, demographic and substance data by evolution groups.

	sCON (Stable-Stable/Stable-Improved)	iCON (Stable-Progressed/Progressed-Stable/Progressed-Improved)	dCON (Progressed-Progressed)	Total	*p* Value
*N*	52	61	32	145	
Age	73.6 ± 3.4	73.9 ± 3.3	74.0 ± 3.8	73.8 ± 3.5	0.898
Gender					0.321
Female	33 (63.5%)	30 (49.2%)	18 (56.3%)	81 (55.9%)	
Male	19 (36.5%)	31 (50.8%)	14 (43.8%)	64 (44.1%)	
Education (year)					0.315
<9	10 (19.2%)	5 (8.2%)	6 (18.8%)	21 (14.5%)	
9–12	20 (38.5%)	29 (47.5%)	16 (50.0%)	65 (44.8%)	
>12	22 (42.3%)	27 (44.3%)	10 (31.3%)	59 (40.7%)	
MMSE	28.6 ± 1.2	28.3 ± 1.3	28.5 ± 1.7	28.5 ± 1.4	0.534
Chocolate (serving/month)	61.3 ± 58.5	56.0 ± 49.2	46.4 ± 44.4	55.8 ± 51.7	0.443
Coffee (cup/month)	56.3 ± 32.6	50.6 ± 36.1	58.7 ± 43.2	54.4 ± 36.5	0.535
Wine (glass/month)	18.6 ± 18.3	28.1 ± 29.9	34.5 ± 43.7	26.1 ± 30.7	0.054
Chocolate (tertile)					0.689
Light	18 (34.6%)	20 (32.8%)	15 (46.9%)	53 (36.6%)	
Moderate	17 (32.7%)	22 (36.1%)	7 (21.9%)	46 (31.7%)	
Heavy	17 (32.7%)	19 (31.1%)	10 (31.3%)	46 (31.7%)	
Coffee (tertile)					0.228
Light	12 (23.1%)	25 (41.0%)	13 (40.6%)	50 (34.5%)	
Moderate	21 (40.4%)	19 (31.1%)	7 (21.9%)	47 (32.4%)	
Heavy	19 (36.5%)	17 (27.9%)	12 (37.5%)	48 (33.1%)	
Wine (tertile)					0.154
Light	24 (46.2%)	17 (27.9%)	12 (37.5%)	53 (36.6%)	
Moderate	19 (36.5%)	30 (49.2%)	8 (25.0%)	57 (39.3%)	
Heavy	9 (17.3%)	14 (23.0%)	12 (37.5%)	35 (24.1%)	

## Supplementary Materials

The following are available online at http://www.mdpi.com/2072-6643/10/10/1391/s1, Figure S1: Timeline of imaging and neuropsychological testing.
