# Supplementary figures and images for: Impact of Coffee, Wine, and Chocolate Consumption on Cognitive Outcome and MRI Parameters in Old Age
Source: Nutrients. 2018 Oct 1;10(10):1391. doi: 10.3390/nu10101391 (PMC6212945; doi:10.3390/nu10101391)

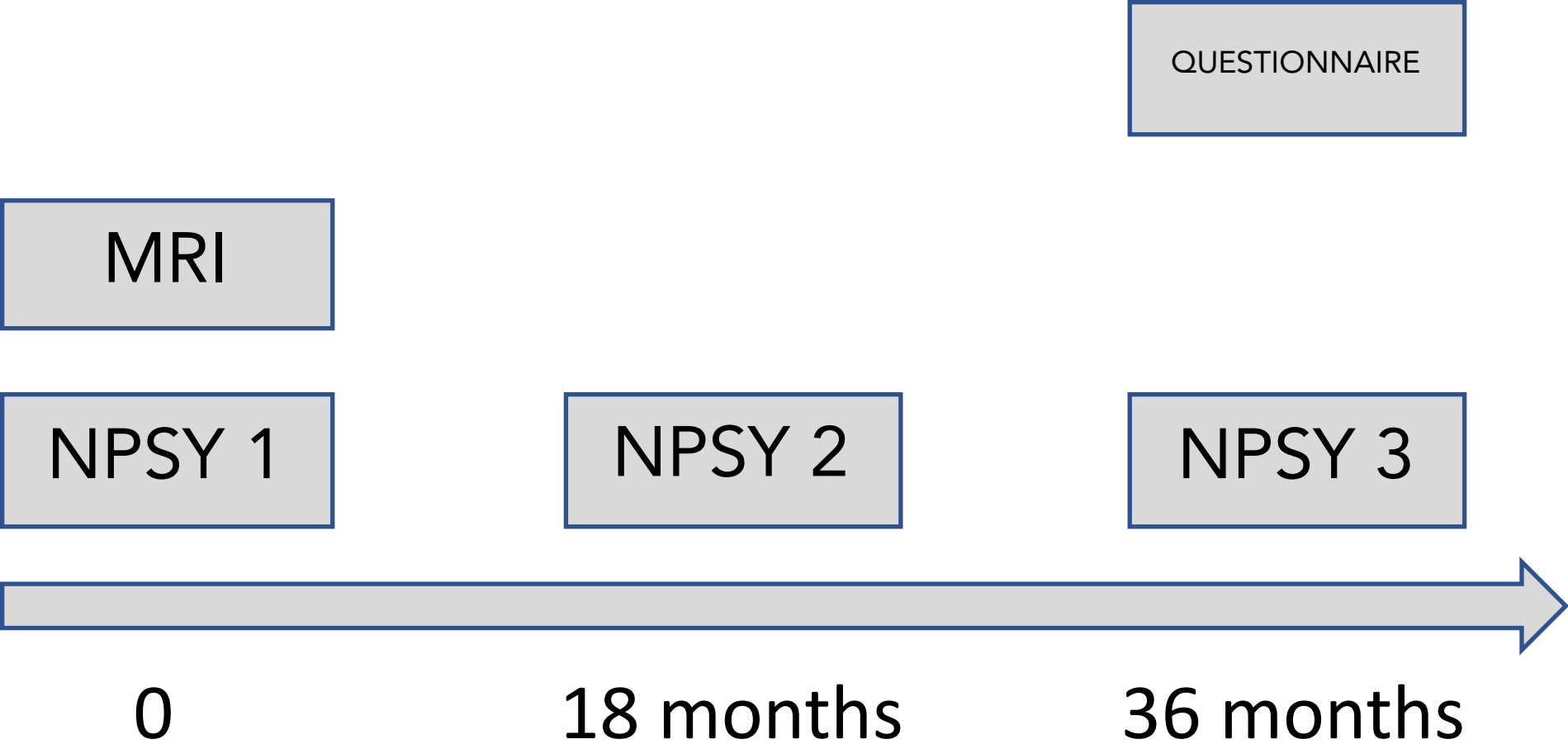

Supplement: Supplementary file 1 [file nutrients-10-01391-s001.pdf]
